# Effective adsorption of amoxicillin by using UIO-66@ Cr-MIL-101 nanohybrid: isotherm, kinetic, thermodynamic, and optimization by central composite design

**DOI:** 10.1038/s41598-023-49393-7

**Published:** 2023-12-20

**Authors:** Soheila Sharafinia, Alimorad Rashidi, Farnoush Tabarkhoon, Fahime Dehghan, Farnaz Tabarkhoon, Mohammad Bazmi

**Affiliations:** 1 Department of Chemistry, Faculty of Science, Shahid Chamran University of Ahvaz, Ahvaz, Iran; 2 Nanotechnology Research Center, Research Institute of Petroleum Industry (RIPI), Tehran, Iran; 3 College of Chemical, Petroleum and Gas Engineering, Iran University of Science and Technology, Tehran, Iran; 4 School of Chemical Engineering, College of Engineering, University of Tehran, Tehran, Iran; 5 Faculty of Chemical Engineering, Amirkabir University of Technology, Tehran, Iran

**Keywords:** Environmental sciences, Environmental social sciences, Chemistry, Materials science, Nanoscience and technology

## Abstract

In this research, the amoxicillin (AMX) removal was studied on a prepared nanosorbent from MOFs. The aim of this research work is to prepare nanohybrids based on metal–organic frameworks (MOFs) as an efficient nanosorbent for the absorption of amoxicillin drug. In this study, UIO-66 nanoparticles (UIO-66 NPs) were prepared from Zirconium (Zr) metal and 1,4-benzene dicarboxylic acid (BDC). Then UIO-66@Cr-MIL-101 nanohybrid was synthesized by hydrothermal method. Structural and physicochemical properties of nanohybrid UIO-66@Cr-MIL-101 were characterized by different analyses such as X-ray diffraction analysis (XRD), fourier transform infrared spectrometer (FT-IR), scanning electron microscopy (SEM), energy dispersive X-ray (EDX), transmission electron microscopy (TEM), therapeutic goods administration (TGA), and Brunauer–Emmett–Teller (BET). The effect of four fundamental variables effective on adsorption was optimized by the central composite response surface methodology (CCRSM). This parameters including loading percentage of Cr-MIL-101 NPs (10–30%), initial concentration of AMX (20–140 mg L^−1^), contact time (20–60 min), and pH (20–10). The removal percentage (Re%) of AMX equal to 99.50% was obtained under the following conditions: The loading value of 20% Wt%, the initial concentration of AMX 80 mg L^−1^, contact time 20 min, and pH = 6. Also, the experimental data were investigated with famous kinetic models and isotherms, and it was observed that AMX removal by nanohybrid is correlated with the PSO kinetic model and Langmuir isotherm.

## Introduction

In recent years, due to the growth of population and various industries, environmental pollution has increased due to drugs and antibiotics, becoming one of the serious and leading problems in the field of environment. Since they are used as a solution for treating diseases and improving health in human societies, the consumption of antibiotics directly or indirectly leads to their entry into the environment. As a result, these chemical substances can remain in the environment for a long time and act as environmental pollutants. In addition, excessive use of drugs and antibiotics can create resistance in bacteria and microorganisms, which can lead to problems such as the emergence of drug-resistant diseases^1^. Removing drugs and antibiotics from the environment and water is a complex challenge that requires scientific and practical solutions. These solutions include: using advanced wastewater treatment systems^2^, using nanotechnology and active NPs, nanofiltration, and nanoabsorbents^3^, using biological activities of bacteria and fungi to decompose drugs^4^, using physical activities and filtration methods, adsorption^5–10^, oxidation and ultrasonic methods^11–15^, providing necessary training to patients and physicians to reduce drug consumption^16^, recycling drugs^17^, removing expired drugs and preventing their entry into the environment^18^, and using ultraviolet radiation and hydrogen peroxide oxidation to reduce and disinfect antibiotic residues^19^. Among these methods, the use of nanoadsorbents for the removal of antibiotics from water has emerged as a new and efficient method for combating water pollution due to the presence of drugs and antibiotics due to their selectivity and high efficiency. Nanoadsorbents are considered smart and highly sensitive absorbents to environmental components. As shown in a report^20^, these absorbents selectively absorb antibiotics from water using their nanoscale properties, instead of using chemical and physical processes for antibiotic removal. Nanoadsorbents, due to their larger surface area compared to larger adsorbents such as activated carbon^21^, for example, can improve the efficiency of antibiotic removal from water adsorption methods. Additionally, due to their limited movement in the environment, nanoadsorbents can selectively absorb antibiotics from water and remove them faster and with higher efficiency^22^.

Currently, many nanoadsorbents have been studied and researched for the removal of antibiotics from water, including metal NPs (silver, iron, and gold NPs)^23^, carbon^24^, silica NPs^25^, and zinc oxide NPs^26^. The use of MOFs as nanoadsorbents for adsorption process has recently gained attention^27–31^. These types of nanoadsorbents are very powerful and, with their structural complexity, can selectively absorb various drugs from water. One of the advantages of using MOFs compared to the aforementioned nanoadsorbents is their ability to regulate their chemical nature. By changing the type of metal used in the construction of MOFs, their adsorption properties can be altered and used for the adsorption of various drugs. Additionally, by changing the type of organic compounds used in the construction of MOFs, their adsorption properties can also be altered^32,33^. So far, different MOFs have been identified and studied as strong adsorbents for various antibiotics^34,35^. The results of Methotrexate adsorption by Cr-MIL-101 NPs from water showed that this nanoadsorbent selectively absorbed Methotrexate from water with the highest efficiency. Additionally, this nanoadsorbent can be reused due to its easy recovery^36,37^. Paracetamol is a type of analgesic used in the treatment of pain and fever and is commonly found in municipal water and wastewater. In a study, UIO-66 NPs were used as a nanoadsorbent for the adsorption of Paracetamol from water, which was effective in selectively adsorbing Paracetamol with high efficiency^38^. Overall, UIO-66 NPs have been identified as a powerful nanoadsorbent for drug adsorption from water and are used for various applications such as drinking water and wastewater. Additionally, combining two MOFs creates a more complex structure with increased internal surface area, allowing larger molecules, such as drugs, to be adsorbed^39^.

The aim of this study is to use the UIO-66@Cr-MIL-101 nanohybrid for the adsorption of the antibiotic AMX (see Table 1). The XRD results confirmed the crystallinity of UIO-66@Cr-MIL-101 nanohybrid, and the particle size of this nanohybrid was between 8 and 110 nm (based on the SEM results). AMX, as a large molecule with high molecular weight, is adsorbed on the internal surface of UIO-66@Cr-MIL-101. The attractive and repulsive forces between the adsorbent's internal surface and AMX cause AMX to be trapped in the UIO-66@Cr-MIL-101 structure. Design-Expert software was used to achieve maximum adsorption and optimize effective parameters for adsorption, and the optimal parameters and experimental results were analyzed for the study of reaction kinetics and thermodynamics. General, UIO-66@Cr-MIL-101 nanohybrid is a class of nanomaterials that have been shown to be effective adsorbents for a variety of pollutants, including amoxicillin. Amoxicillin is a widely used antibiotic, but it can also be a contaminant in wastewater and surface water. Adsorption by UIO-66@Cr-MIL-101 nanohybrid has several advantages over other methods of amoxicillin removal, including: (1) high adsorption capacity: UIO-66@Cr-MIL-101 nanohybrid have a high surface area and porosity, which allows them to adsorb large amounts of amoxicillin. (2) selectivity: UIO-66@Cr-MIL-101 nanohybrid can be functionalized to selectively adsorb amoxicillin from other pollutants in water. (3) regeneration: UIO-66@Cr-MIL-101 nanohybrid can be regenerated and reused, making them a more sustainable option than other adsorbents. In addition to these advantages, UIO-66@Cr-MIL-101 nanohybrid are also relatively inexpensive and easy to produce. This makes them a promising technology for the removal of amoxicillin from wastewater and surface water. Overall, UIO-66@Cr-MIL-101 nanohybrid are a promising technology for the removal of amoxicillin from wastewater and surface water. They have a high adsorption capacity, selectivity, and can be regenerated and reused.

**Table 1:** The physiochemical properties of the AMX.

Molecular structure	
Molecular formula	C_16_H_19_N_3_O_5_S
Molecular weight (g mol^−1^)	365.4
λ_max_ (nm)	270

## Experimental section

### Materials

The substances used in this study were purchased from Merck (Darmstadt, Germany) as follows: Zirconium (IV) chloride (ZrCl_4_), BDC, Chromium nitrate nonahydrate (Cr (NO_3_)_3_⋅9H_2_O, ammonium fluoride (NH_4_F), absolute methanol (MeOH (99.0%)), absolute ethanol (EeOH (99.0%)), absolute N,N-dimethylformamide (DMF (97.0%)), absolute hydrofluoric acid (HF (40%)), and distilled water (DW).

### Preparation of UIO-66 NPs

The UiO-66 NPs were prepared using the reported procedure by Michael et al.^40^.

#### Solution 1

ZrCl_4_ (0.125 g), DMF (5 ml), and concentrated HCl (1 ml) were added to a 10-dram glass vial, and the mixture solution was sonicated for 25 min.

#### Solution 2

BDC linker (0.123 g) and DMF (10 ml) were added into another vial, and then the mixture was stirred until to completely dissolving of the BDC linker.

**Figure 1:**
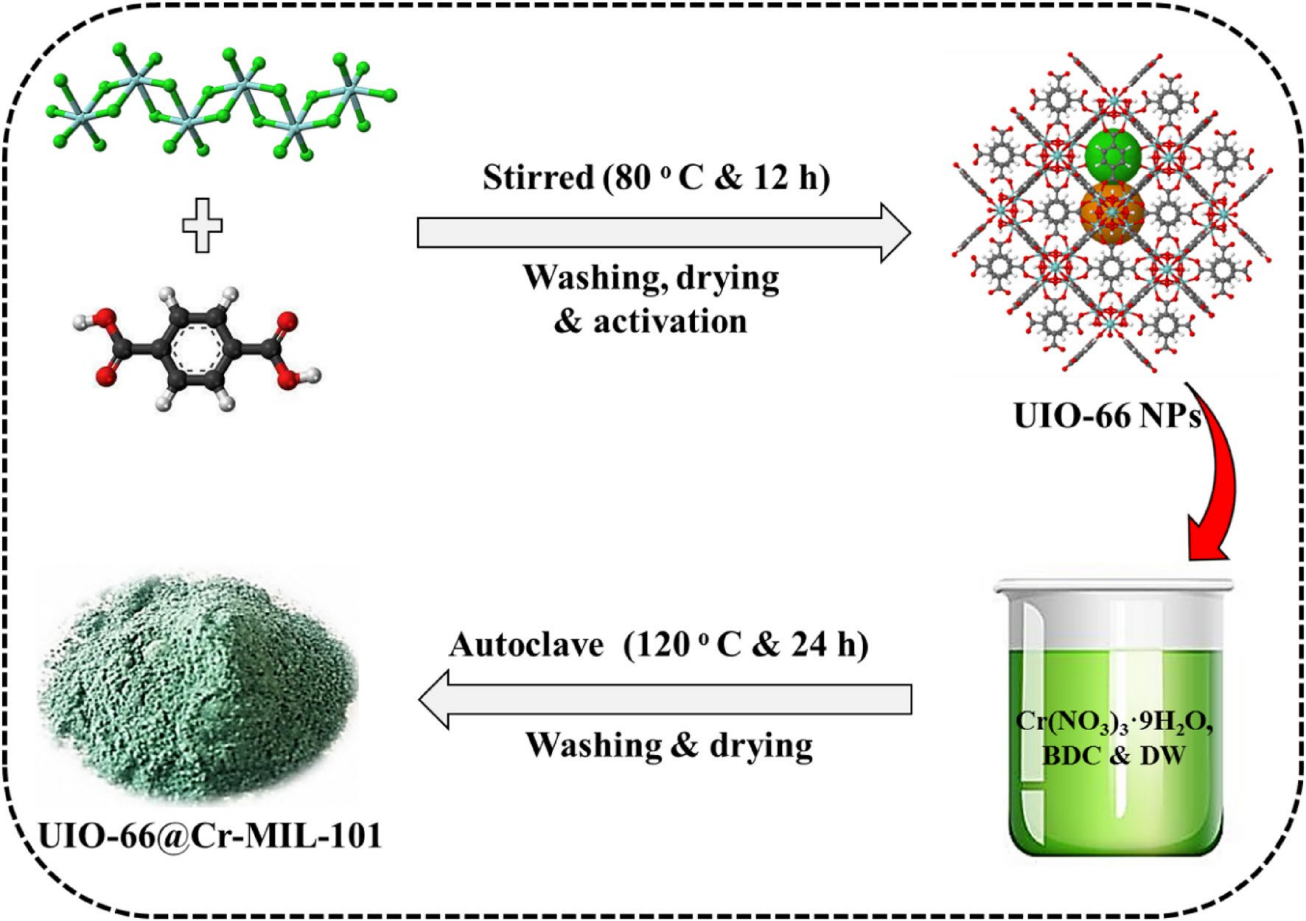
Schem image of synthesis of UIO-66@Cr-MIL-101 nanohybrid.

Then solution 2 was added to solution 1 and was sonicated for 20 min. The resulting mixture was stirred for 12 h at 80 °C. The obtained milky solution was cooled to room temperature, and its precipitate was separated by centrifugation. The precipitate was washed three times with DMF and two times with EtOH. The resulting white UIO-66 NPs are dried in an oven under 80 °C and then activated in a vacuum oven under a temperature of 150 °C for 24 h (Fig. 1).

### Preparation of UIO-66@Cr-MIL-101 nanohybrid

UIO-66@Cr-MIL-101 nanohybrid were synthesized by hydrothermal method, UIO-66 NPs (1 g), Cr (NO_3_)_3_⋅9H_2_O (8 g), and BDC (3.31 g) were added to 95 ml of DW and stirred for 25 min. Then, 20 mmol of HF was added to the mixture, and it was vigorously stirred for another 20 min. The resulting suspension was transferred to the autoclave and kept in the oven for 24 h at 220 °C. The obtained green product was cooled to room temperature and centrifuged for the washing process (Fig. 1).

### Post-synthesis and purification

To purify the UIO-66@Cr-MIL-101 nanohybrid samples from unreacted BDC species, the sample was washed as follows: firstly, the samples were washed with DMF (three times, at 80 °C) and with DW (five times, at 100 °C) and centrifuged. Then the samples were refluxed for 8 h in DMF at 80 °C and separated by centrifuge after cooling. The resulting product was soaked in MeOH for four days at 25 °C (Note: MeOH was regularly changed every day). After that, the powders were activated in a vacuum oven at 140 °C for 12 h. Finally, to remove the residual BDC, the samples were stirred in NH_4_F aqueous solution at 65 °C for 5 h (NH_4_F amount: 100 cm^3^ of 30 mM solution per 1 g of dried powder in the previous step). To remove any remaining NH_4_F molecules inside the pores, UIO-66@Cr-MIL-101 nanohybrids were washed three times with DW (100 °C) and centrifuged. The obtained UIO-66@Cr-MIL-101 nanohybrid was activated for 12 h in a vacuum oven at 140 °C. The prepared product is denoted as UIO-66@Cr-MIL-101 10% nanohybrid. The preparation of other sorbents of A (15–30%) was carried out with this method but with different amounts of Zn and BDC.

### Batch adsorption experiments

The batch adsorption method was used to investigate the removal of AMX by UIO-66@Cr-MIL-101 nanohybrid, study the equilibrium isotherms, kinetics, and thermodynamics. According to the conditions of 30 runs proposed by CCRSM, concentration of initial AMX, pH, contact time and temperature variables were examined. A shaker incubator was used to adsorption experiments at identified temperature and 175 rpm. The adsorption amount of the AMX was measurement by UV–Vis instrument at 270 nm.

AMX R_e_% and equilibrium adsorption capacity (q_e_ (mg g^−1^)) were calculated under different experimental conditions including initial concentration of AMX, pH, contact time and temperature. The R_e_% and q_e_ of AMX is calculated using Eqs. (1) and (2), respectively:

1 $$\text{Re\%}=\frac{{{\text{C}}}_{0}-{{\text{C}}}_{{\text{e}}}}{{{\text{C}}}_{0}}\times 100$$

2 $${{\text{q}}}_{{\text{e}}}= \frac{({{\text{C}}}_{0}-{{\text{C}}}_{{\text{e}}}){\text{V}}}{{\text{m}}}$$

where C_0_ (mg g^−1^), C_e_ (mg g^−1^), V (l), and m (g) are the initial and equilibrium concentrations of AMX, volume of the solution, and the adsorbent, respectively.

## Results and discussion

**Figure 2:**
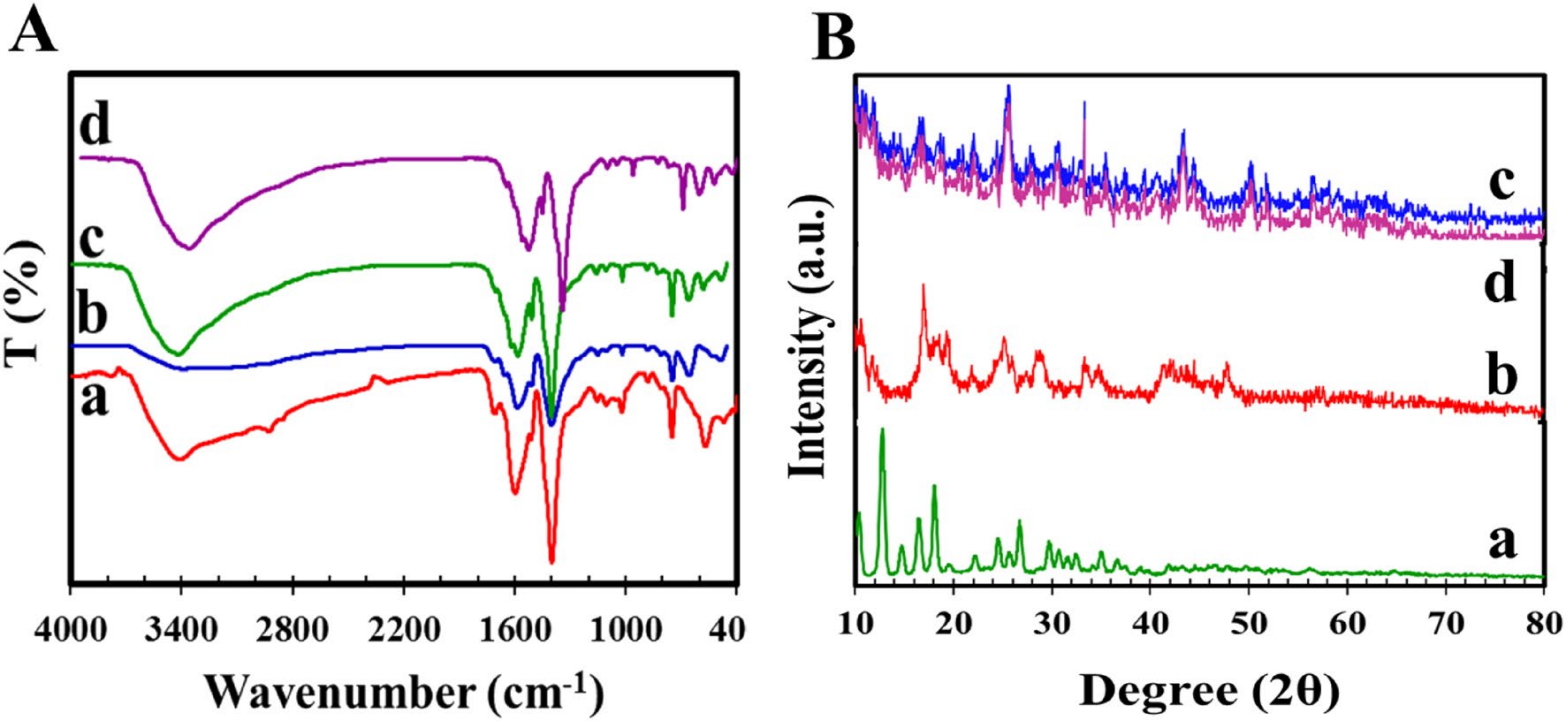
(**A**) FT-IR results of (a) UIO-66 NPs, (b) Cr-MIL-101 NPs, (c) UIO-66@ Cr-MIL-101 nanohybrid, (d) after AMX adsorption; (**B**) XRD pattern of (a) UIO-66 NPs, (b) Cr-MIL-101 NPs, (c) UIO-66@Cr-MIL-101 nanohybrid, (d) after AMX adsorption.

Figure 2A shows the FT-IR spectra of Cr-MIL-101 NPs, UIO-66 NPs, and UIO-66@Cr-MIL-101 nanohybrid. For the UIO-66@Cr-MIL-101 nanohybrid, compared to the other two nanoadsorbents, the spectrum at around 486 cm^−1^ is related to the asymmetric stretching vibration of Zr–OC, the spectrum at around 590 cm^−1^ is related to the Cr–O stretching vibration, and the spectrum at around 700 cm^−1^ is indicative of the C–H stretching vibration of the BDC organic ligand^41,42^. Additionally, the spectrum in the range of 1200–1000 cm^−1^ is attributed to the nitrogen and carbonyl groups in the organic ligand^41^. The symmetric carboxyl group stretching of BDC ligand can be observed in the spectrum at around 1400 cm^−1^. The spectra at around 1600 and 1700 cm^−1^ are indicative of the asymmetric stretching vibration of O–C–O and the carboxyl group –COO– in the organic ligand, respectively^21^. The O–H stretching vibration band (at around 3421 cm^−1^) of the UIO-66 NPs in the UIO-66@Cr-MIL-101 nanohybrid confirms the loading of UIO-66 NPs in the Cr-MIL-101 NPs^39,43^. To verify adsorption mechanisms, the FT-IR spectra of UIO-66@Cr-MIL-101 nanohybrid were compared before and after adsorption. As shown in Fig. 2A-d, the band peak of hydroxyl shifted from 3421 to 3361.92 cm^−1^ after adsorption. The adsorption peak changing from 1569 to 1565 cm^−1^ was stretching vibration of C–O in the carboxyl group^44^, which indicated hydrogen bonding interaction between O in UIO-66@Cr-MIL-101 nanohybrid and H in AMX.

The XRD patterns in Fig. 2B show that the diffraction patterns of UiO-66 NPs, Cr-MIL-101 NPs, and UIO-66@Cr-MIL-101 nanohybrids are very similar and consistent with published data^45,46^. The maximum diffraction peak for the UIO-66@Cr-MIL-101 nanohybrid sample is at 2θ = 44° (see Fig. 2B), while for UiO-66 NPs and Cr-MIL-101 NPs, it is observed at 2θ = 43.5° and 2θ = 42.3°, respectively. The presence of this peak indicates the successful preparation of UiO-66 NPs and a successful combination with Cr-MIL-101 NPs, as shown in Fig. 2B-a,B-b. The XRD pattern of the sample shows typical peaks at 2θ = 9.9°, 18°, and 24.2°, which confirm the successful synthesis of UIO-66 NPs. These peaks were observed in the hybrid sample at θ = 10.9°, 2θ = 17.47°, and 2θ = 24.9°, respectively, indicating that the topology structure of UIO-66 NPs was not significantly affected and that UIO-66 NPs was well combined with Cr-MIL-101. In addition, the XRD pattern of the UIO-66@Cr-MIL-101 nanohybrid shows that there is no change in the main peaks of the binary. However, the intensity of the peaks in the UIO-66@Cr-MIL-101 nanohybrid is significantly reduced compared to UiO-66 NPs, which can be attributed to the good distribution of UIO-66 NPs in the Cr-MIL-101 NPs sample^43^.

Furthermore, according to the XRD of the UIO-66@Cr-MIL-101 nanohybrid after the adsorption process, no specific changes were observed, that confirms the stability of the sample (Fig. 2B).

**Figure 3:**
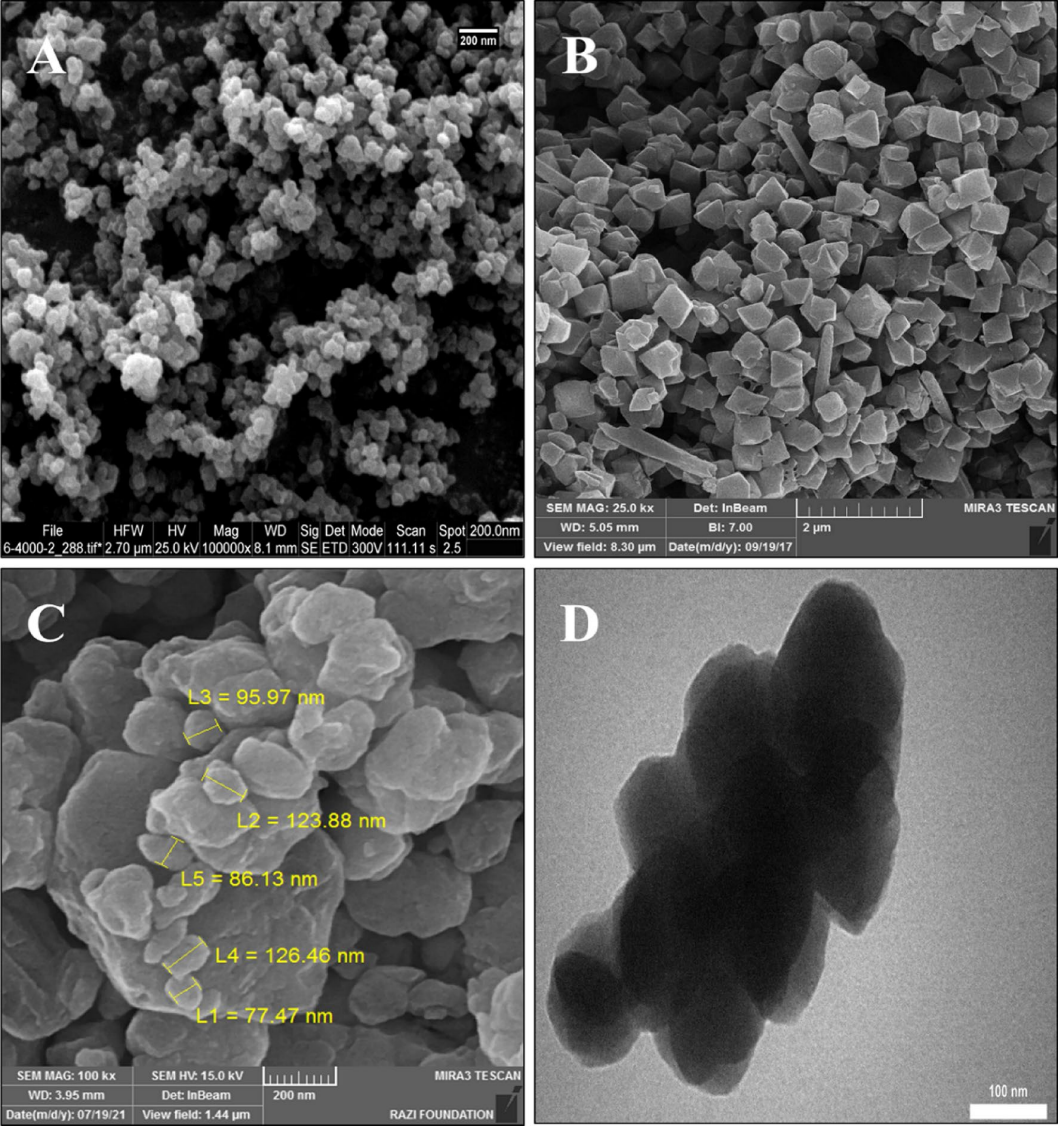
SEM images of (**A**) UIO-66 NPs, (**B**) Cr-MIL-101 NPs, (**C**) UIO-66@Cr-MIL-101 nanohybrid; (**D**) TEM image UIO-66@Cr-MIL-101 nanohybrid.

**Figure 4:**
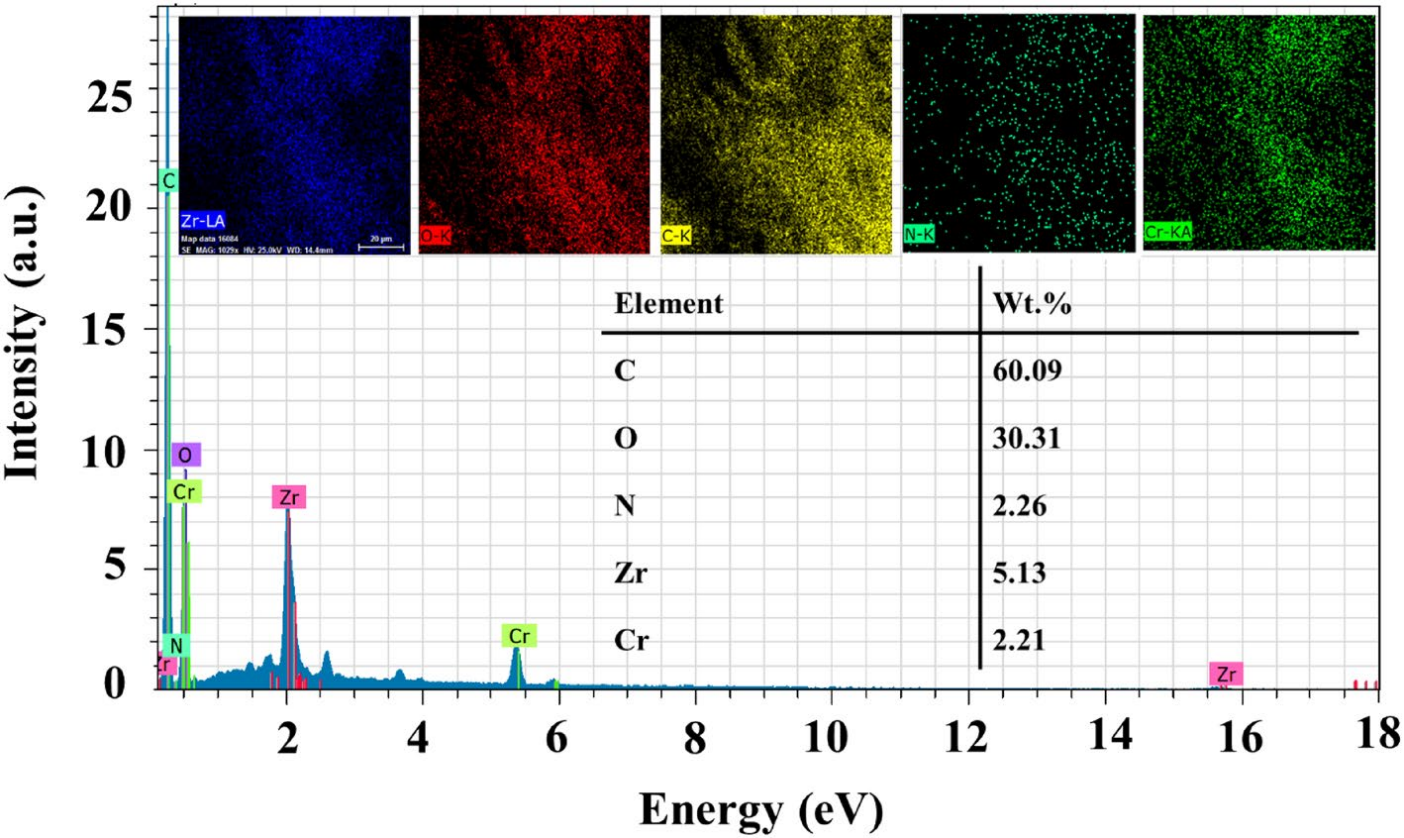
EDX analysis and elemental mapping images of UIO-66@Cr-MIL-101 nanohybrid.

The morphology of the UIO-66 NPs, Cr-MIL-101, and UIO-66@Cr-MIL-101 nanohybrid structures is shown in Fig. 3. As seen in Fig. 3A, UIO-66 NPs crystals have homogeneous and uniform cubic shapes with edges of about 50 nm. Figure 3B shows nanocrystals of Cr-MIL-101 with a regular geometrical shape of a polyhedron, approximately 400 nm in size, and with sharp and non-intersecting corners. In Fig. 3C, the Cr-MIL-101 NPs and UIO-66 NPs crystals are integrated, and the UIO-66 NPs include the regular crystals of Cr-MIL-101, changing their geometrical shape so that the regular geometrical shape is no longer visible. As evident from Fig. 3D, the TEM image shows the porous network of the UIO-66@Cr-MIL-101 hybrid, which includes the hierarchical structure of the UIO-66 NPs crystals and the Cr-MIL-101 NPs crystals. At the same time, the elements C, O, Fe, Si, and Zr were directly observed from the EDX pattern of the UIO-66@Cr-MIL-101 nanohybrid in Fig. 4. The presence of iron and silicon elements was helpful evidence that showed the successful preparation of the nanohybrid core–shell structure^47^.

**Figure 5:**
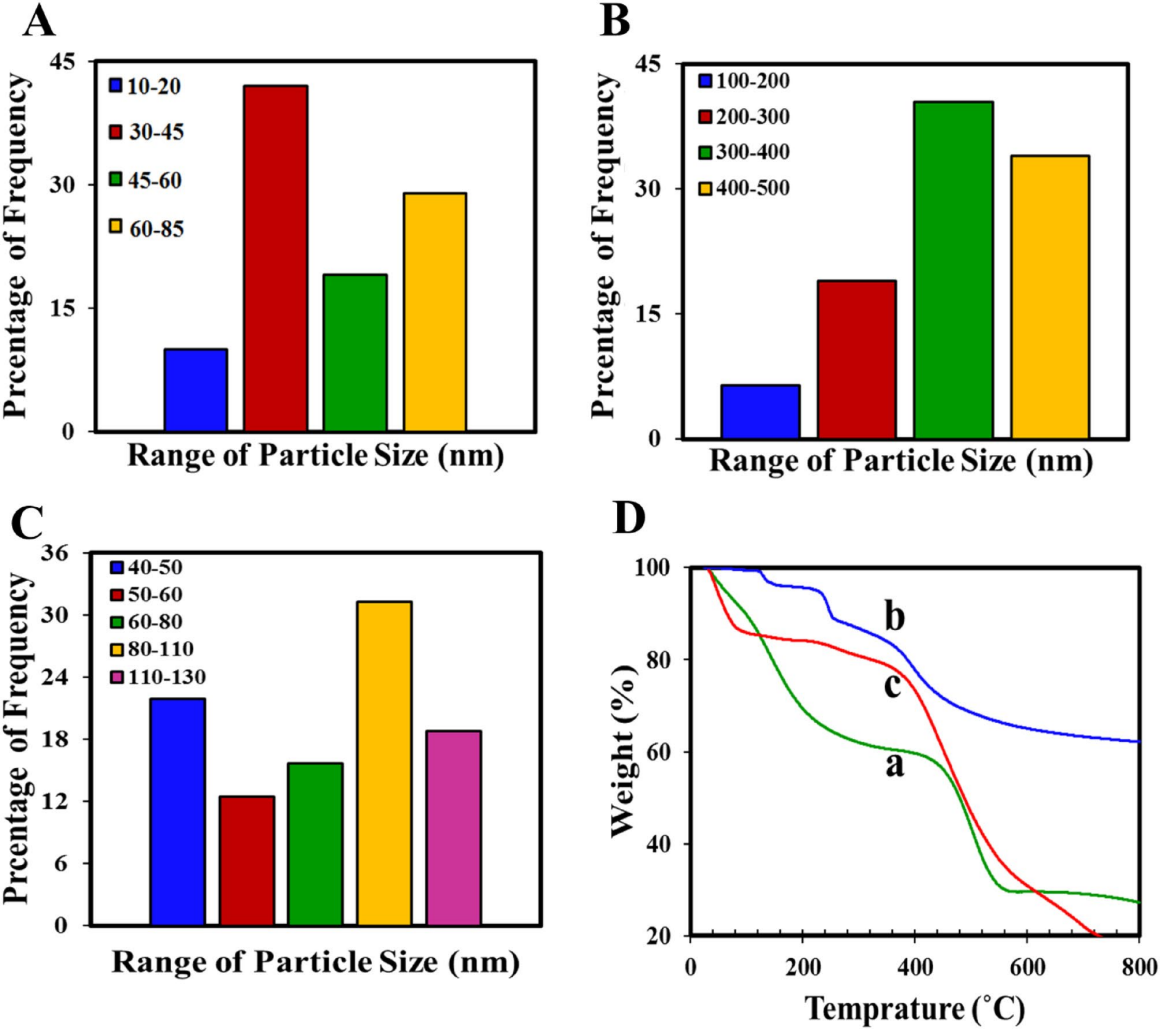
PSD curves of (**A**) UIO-66 NPs, (**B**) Cr-MIL-101 NPs, (**C**) UIO-66@Cr-MIL-101 nanohybrid; (**D**) TGA curves of (a) UIO-66 NPs, (b) Cr-MIL-101 NPs, (c) UIO-66@ Cr-MIL-101 nanohybrid.

The particle size distribution (PSD) curve (Fig. 5A) shows a narrow and focused distribution of the sizes of the UIO-66 NPs pores around a specific value (the highest percentage of PSD is in the range of 30–45 nm). This is because the synthesis of these NPs usually results in a relatively uniform distribution of pore sizes, which can be used as channels for the transport of molecules or channels for the adsorption or combination of different molecular species. As evident from Fig. 5B,C, the highest percentage of PSD for Cr-MIL-101 NPs and the UIO-66@Cr-MIL-101 nanohybrid is approximately 300–400 nm and 80–110 nm, respectively, which confirms that the pore sizes in the hybrid sample are almost the average pore size in Cr-MIL-101 NPs and UIO-66 NPs, and therefore, the successful preparation of the nanohybrid has resulted in a more desirable PSD and the creation of relatively smaller and uniform crystals.

The thermal behaviors of three samples, UIO-66 NPs, Cr-MIL-101, and UiO-66@Cr-MIL-101, were studied using TGA. From the TG curves (Fig. 5D), it is evident that both UIO-66 NPs and Cr-MIL-101 NPs exhibit three weight loss stages. The first weight loss between 30 and 100 °C is due to the evaporation of absorbed water in the MOF structure, while the second weight loss stage between 100 and 320 °C is attributed to the removal of DMF molecules. The third weight-loss stage begins at 450 °C and is associated with the combustion of the organic ligand, which leads to the decomposition of both MOFs and their composite. Furthermore, the UiO-66@Cr-MIL-101 nanohybrid lost 52.7% of its total weight, indicating its higher thermal stability compared to UIO-66 NPs and Cr-MIL-101, which had a total weight loss of 56.5%^45^.

**Figure 6:**
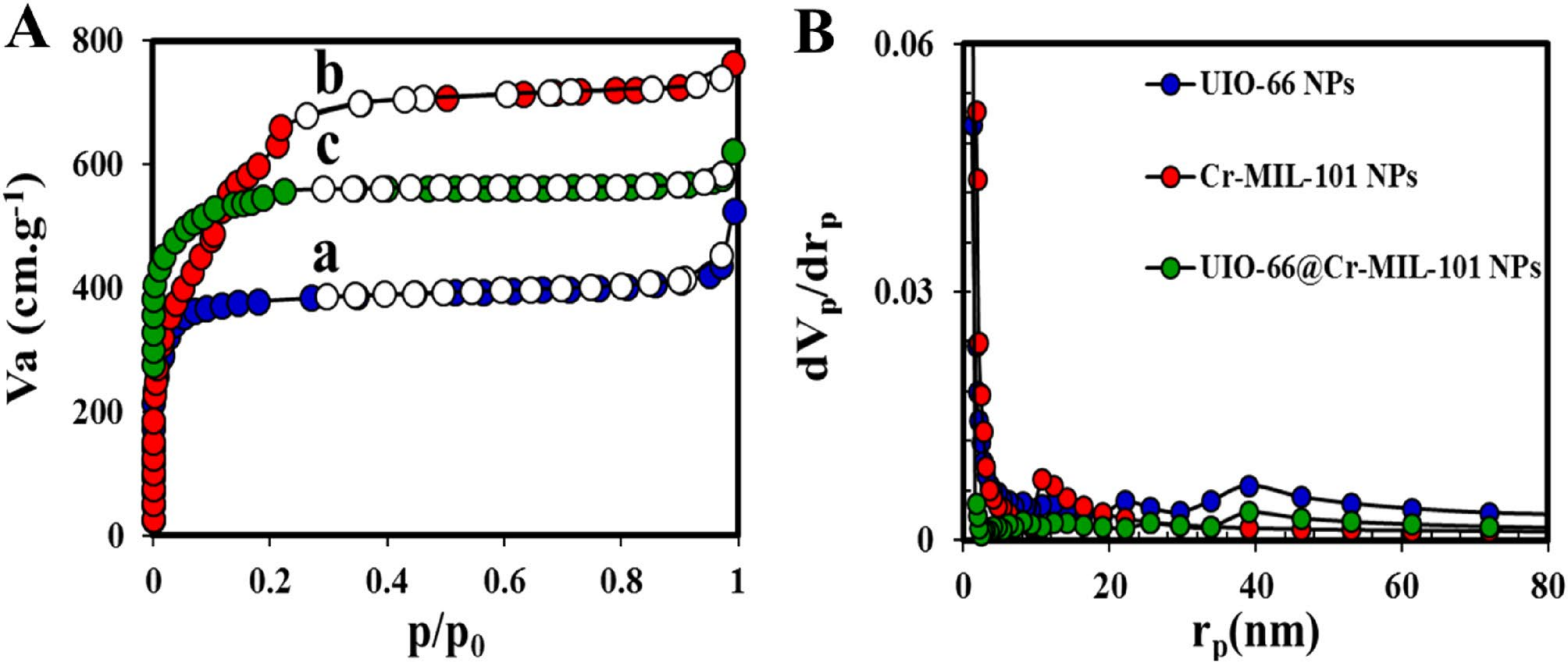
(**A**) N_2_ adsorption/desorption isotherms of (a) UIO-66 NPs, (b) Cr-MIL-101 NPs, (c) UIO-66@ Cr-MIL-101 nanohybrid; (**B**) BJH curves of UIO-66 NPs, Cr-MIL-101 NPs, and UIO-66@ Cr-MIL-101 nanohybrid.

Figure 6A shows the nitrogen adsorption/desorption isotherms of UIO-66 NPs, Cr-MIL-101, and UIO-66@Cr-MIL-101 at a temperature of 77 K. As evident from the figure, all three isotherms were of type I. As shown in Fig. 6A, for all three prepared samples, the adsorption capacity starts with relatively low pressure and rapidly increases with a gradual increase in pressure, which ends at high pressure. This behavior confirms the nature of the porous microstructure in the samples. The specific surface area of UIO-66 NPs was evaluated to be 1350.67 m^2^ g^−1^ (Table 2), which is similar to what has been evaluated in previous studies^29^. However, the surface area and pore volume in UIO-66@Cr-MIL-101 were 1025.30 m^2^ g^−1^ and 0.4441 cm^3^ g^−1^, respectively, both slightly lower than UIO-66 NPs. This reduction could be the result of the encapsulation of Cr-MIL-101 NPs inside UIO-66 NPs. Based on the IUPAC classification, pore diameters are divided into three categories: microspore (the pore size < 2 nm), mesopore (the pore size 2–50 nm), and macrospore (the pore size > 50 nm)^48–51^. Since the diameter pores of the synthesized UIO-66@Cr-MIL-101 nanohybrid is about 2 nm, it has a microporous structure. Additionally, the BJH plot (Fig. 6B) also clearly shows that the pore size distribution in the UIO-66@Cr-MIL-101 nanohybrid is narrower than that of Cr-MIL-101 NPs and UIO-66 NPs, and the majority of the crystal sizes are in the range of 1–20 nm with a maximum of 80 nm^52^.

**Table 2:** Textural properties parameters of UIO-66 NPs, Cr-MIL-101 and UIO-66@ Cr-MIL-101.

Samples	BET specific surface area (m^2^ g^−1^)	Total pore volume (cm^3^ g^−1^)	Average pore diameter (nm)
UIO-66 NPs	1505	0.78	2.12
Cr-MIL-101	3440	1.82	1.37
UIO-66@Cr-MIL-101	1921.70	0.56	2.02

### CCRSM

The response surface methodology (RSM) is used to evaluate the correlation between experimental data and the outcomes attained. This particular method is a statistical technique that analyzes multiple variables simultaneously, aiming to enhance experimental procedures, optimize results, and minimize the need for excessive experimentation. The optimization process involves several steps, including: (1) carrying out statistically planned tests, (2) evaluating the coefficients within a mathematical model, and (3) identifying the response and assessing the model's accuracy^53^. Central composite design (CCD) has various applications, for example, adsorption processes^54^, chromatographic methods^55^, and spectroanalytical methods^56^. In this research study, the CCD method implemented through Design Expert 11.0.3.0 software is utilized to introduce randomness in the experimental runs, design the experiments, study the significant impacts of operational factors on AMX removal, and identify a combination of variables that maximize the efficiency of AMX adsorption. Randomization is essential to ensure that the outcome of each run is independent of the others, thereby obtaining accurate experimental results. To optimize the parameters, a quadratic model is employed to establish a relationship between the selected variables and the response. Equation (3) represents the quadratic equation model used in this context^57^.

3 $${\text{Y}}\left(\text{Re\%}\right)={\upbeta }_{0}+\sum_{{\text{i}}=1}^{{\text{k}}}{\upbeta }_{{\text{i}}}{{\text{X}}}_{{\text{i}}}+\sum_{{\text{i}}=1}^{{\text{k}}}{\upbeta }_{{\text{ii}}}{{\text{X}}}_{{\text{i}}}^{2}+\sum_{{\text{i}}=1}^{{\text{k}}}\sum_{{\text{j}}=1}^{{\text{k}}}{\upbeta }_{{\text{ij}}}{{\text{X}}}_{{\text{i}}}{{\text{X}}}_{{\text{j}}}+{{\text{e}}}_{0}$$

In the given equation, Y (Re%) represents the response variable, while β_0_, β_i_, β_ii_, and β_ij_ correspond to the constant coefficient, linear coefficient, quadratic coefficient, and cross-product coefficient (interaction coefficient), respectively. Additionally, X_i_ and X_j_ are coded variables, and to determine their values, one can utilize multiple regression analysis as described in Eq. (4)^58^:

4 $$ {\text{X}}_{{\text{i}}}  = \frac{{{\text{X}}_{{\text{i}}}  - {\text{X}}_{0} }}{{\updelta {\text{X}}}} $$

where X_0_ and X_i_ (at the center point) denote the actual values of the independent variable, while δX refers to the incremental changes between the low (− 1) and high (+ 1) levels.

### Optimization of adsorption of AMX onto UIO-66@Cr-MIL-101 nanohybrid using CCRSM

In this study, four crucial factors were investigated: the loading of MIL-101 NPs on UIO-66 NPs (A = 10–30 Wt%), the initial concentration of AMX (B = 20–140 mg L^−1^), pH (C = 20–10), and contact time (D = 20–60 min) (refer to Table 3). Additionally, utilizing the CCRSM approach, a total of 30 experimental runs were conducted to assess the impact of these independent variables on the adsorption efficiency of AMX using the UIO-66@Cr-MIL-101 nanohybrid. The average results of both experimental and predicted outcomes for this process are presented in Table 4. Furthermore, the central point parameters were also replicated six times to establish the reproducibility of the experiments and confirm the consistency of the obtained data.

**Table 3:** Independent variables and levels of the process for CCRSM.

Independent variables	Symbol	Levels of independent variables				
		− α	− 1	0	+ 1	+ α
Loading (Wt%)	A	10	15	20	25	30
Concentration (mg L^−1^)	B	20	50	80	110	140
pH	C	2	4	6	8	10
Time (min)	D	20	30	40	50	60

**Table 4:** Independent variables and levels of the process for BBRSM.

Run	A	B	C	D	UIO-66@Cr-MIL-101 nanohybrid	
					Re% (actual)	Re% (predicted)
1	30	80	6	40	97.31	96.7
2	10	80	6	40	78.86	78.38
3	20	80	10	40	70.8	72.4
4	15	50	4	50	95.28	95.27
5	15	110	4	50	72.06	72.61
6	15	110	8	50	59.11	61.78
7	20	140	6	40	60.67	57.75
8	20	80	2	40	82.76	80.07
9	20	80	6	40	82.76	81.39
10	15	110	8	30	66.86	67.48
11	25	50	4	50	95.49	95.83
12	20	80	6	40	79.9	81.39
13	20	20	6	40	88.2	90.03
14	20	80	6	40	81.2	81.39
15	20	80	6	40	79.12	81.39
16	25	110	4	30	79.05	82.83
17	15	110	4	30	62.32	61.88
18	20	80	6	60	92.37	92.46
19	20	80	6	20	95.23	94.05
20	15	50	4	30	84.26	87.33
21	25	110	8	30	85.1	85.24
22	15	50	8	50	88	85.19
23	25	50	8	30	94.45	94.86
24	20	80	6	40	82.76	81.39
25	20	80	6	40	82.63	81.39
26	25	50	4	30	94.24	91.7
27	15	50	8	30	95.28	93.69
28	25	110	4	50	88.03	89.75
29	25	50	8	50	81.98	82.55
30	25	110	8	50	77.82	75.72

### Analysis of variant (ANOVA)

For AMX uptake using UIO-66@Cr-MIL-101 nanohybrid, different models were examined, quadratic, 2FI, linear, mean, and cubic models. The best model was quadratic to evaluate the data for UIO-66@Cr-MIL-101 nanohybrid. There is also a significant and excellent correlation between the adjusted R^2^ (i.e., 0.94) and predicted R^2^ (i.e., 0.85), which proves the compatibility of available data with new observations (Table 5). As a rule, a difference of more than 0.2 between the adjusted R^2^ and predicted R^2^ would render the model statistically insignificant. In this study, this variation is less than 0.2 for UIO-66@Cr-MIL-101 nanohybrid; thus, the chosen model has high accuracy^59^.

**Table 5:** Model summary statistics for AMX adsorption response by UIO-66@Cr-MIL-101 nanohybrid.

UIO-66@Cr-MIL-101 nanohybrid			
Source	Sequential p-value	Adjusted R^2^	Predicted R^2^
Linear	< 0.0001	0.5681	0.4381
2FI	0.0310	0.7218	0.6522
Quadratic	**< 0.0001**	**0.9463**	**0.8563**
Cubic	0.0441	0.9690	

Significant values are in bold.

The importance of each coefficient and the degree of interaction among each independent variable can be determined by examining the p-value and F-value, respectively. In terms of the model's parameters, it is crucial for the F-value to exceed one and for the p-value to be below 0.05^60^. An Analysis of Variance (ANOVA) was performed on the AMX adsorption using the UIO-66@Cr-MIL-101 nanohybrid, and the results are presented in Table 6. The table demonstrates that the selected models employed to investigate the AMX adsorption process with the UIO-66@Cr-MIL-101 nanohybrid were statistically significant, as indicated by the low p-values (e.g., < 0.0001). Additionally, the substantial F-value further supports the validity of the model and suggests that the experimental systems can be accurately represented with minimal error (Table 6).

**Table 6:** Analysis of variance for the modified quadratic.

Source	Sum of squares	df	F-value	p-value	
Model	3216.95	9	55.03	< 0.0001	Significant
A	501.00	1	77.45	< 0.0001	
B	1441.26	1	240.71	< 0.0001	
C	85.21	1	13.6	0.0015	
AB	262.82	1	42.33	< 0.0001	
CD	265.23	1	41.61	< 0.0001	
A^2^	63.57	1	9.97	0.005	
B^2^	97.55	1	14.86	0.001	
C^2^	47.65	1	7.02	0.0154	
D^2^	248.35	1	37.15	< 0.0001	
Residual	129.9	20			
Lack of fit	117.21	15	3.08	0.1097	Not significant
Pure error	13.00	5			
Cor total	3352.79	29			

The effective value of each parameter, regression coefficients, standard effect values, and standard errors are shown in Table 7.

**Table 7:** The ANOVA results of the response surface modified quadratic model.

UIO-66@Cr-MIL-101 nanohybrid			
Std. Dev.	2.55	R^2^	0.961
Mean	83.00	Adjusted R^2^	0.943
C.V. %	3.09	Predicted R^2^	0.9470
PRESS	340.10	Adeq precision	0.898
− 2 Log Likelihood	129.10	BIC	163.11
		AICc	160.68

### Diagnostic model

To validate the accuracy of the proposed model data, an alternative method involves evaluating the normality of the data, ensuring their adherence to a normal distribution.

**Figure 7:**
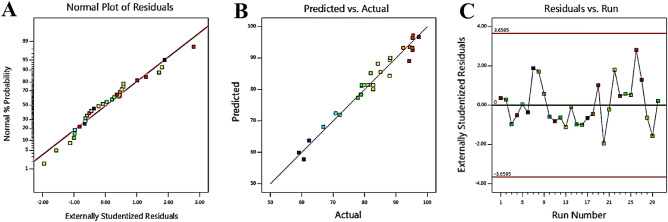
The curve of (**A**) the normal probability; (**B**) the predicted response versus actual response; (**C**) analysis of residual for the response to the UIO-66@Cr-MIL-101 nanohybrid.

Figure 7A presents the normal values and measured statistics of the suggested model for the UIO-66@Cr-MIL-101 nanohybrid. The obtained curve indicates that the collected data for the UIO-66@Cr-MIL-101 nanohybrid is closely near the straight line, showing a normal distribution and further supporting the selection of the model. Furthermore, Fig. 7B includes the outcomes of the mathematical model and experimental data for AMX adsorption by UIO-66@Cr-MIL-101 nanohybrid. The obtained data validate the accuracy of the suggested model.

Residual analysis is employed for the identification and prediction of the suggested model's response^61^. The remaining equal distribution in the adsorbed value range indicates that a substantial variation in the experimental results is primarily attributed to the displacement. Consequently, the suggested model is deemed valid and successfully elucidates the adsorption process (Fig. 7C).

### Response surface analysis to UIO-66@Cr-MIL-101 nanohybrid

**Figure 8:**
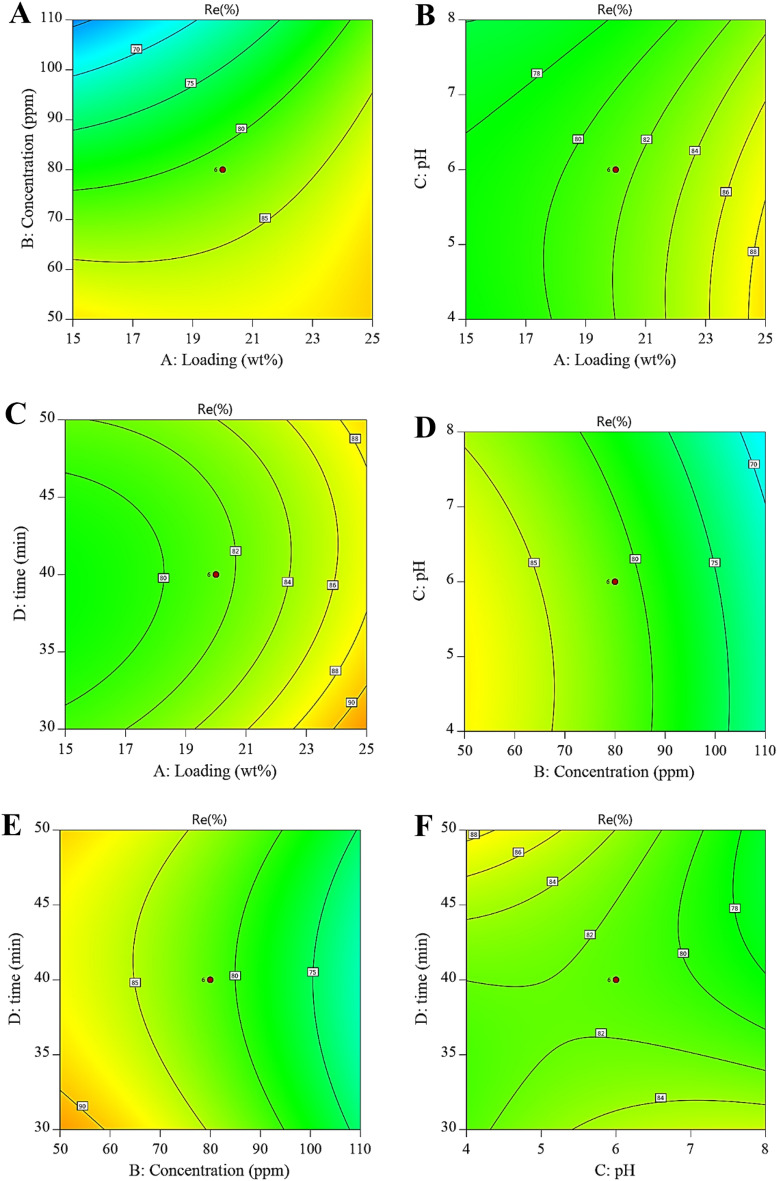
Cantour plots of AMX adsorption onto UIO-66@Cr-MIL-101 nanohybrid: (**A**) the effect of loading and concentration; (**B**) the effect of loading and pH; (**C**) the effect of loading and time; (**D**) the effect of concentration and pH; (**E**) the effect of concentration and time; (**F**) the effect of pH.

Figure 8 shows the cantour plots of the effects of the variables of Cr-MIL-101 NPs loading percentage, initial concentration of AMX, pH, and contact time against the Re%. The red region indicates where the adsorption is maximum (hot zone), graduating through yellow and green to blue zones, where the adsorption is minimal (cold zone). As can be seen from Fig. 8A,D,E, with increasing initial concentration (80 mg L^−1^), the Re% increases. After reaching the equilibrium point, the Re% is almost constant because at high concentrations the active sites are saturated, which leads to repulsion between the adsorbent and the AMX molecules^62^. Figure 8B,D,F show the effect of pH on AMX removal by UIO-66@Cr-MIL-101 nanohybrid. The Re% of AMX increases slightly with increasing pH. The highest Re% of AMX was obtained at pH = 6, which related to the pHpzc of the nanohybrid (the pHpzc of the UIO-66@Cr-MIL-101 nanohybrid was 4.1). In acidic pH, the surface of UIO-66@Cr-MIL-101 nanohybrid has more positive charge, so the attraction between these charges with the negative charge of AMX leads to an increase in Re%. The reduction of Re% in higher pH (as indicated by the blue region) is related to the competition between AMX anions and OH^−^ ions on the surface of the UIO-66@Cr-MIL-101 nanohybrid. However, at basic pHs, the Re% of AMX is acidic due to the opposite charge of the nanohybrid surface and AMX molecules (Fig. 8)^63^.

The mechanism of AMX adsorption by UIO-66@Cr-MIL-101 nanohybrid involves a combination of different factors, including: (1) electrostatic interactions: AMX is a zwitterionic molecule, meaning that it has both positive and negative charges. The metal ions in UIO-66@Cr-MIL-101 can interact electrostatically with the charged groups on AMX, helping to retain the antibiotic on the MOF surface. (2) Hydrogen bonding: AMX also has several functional groups that can form hydrogen bonds with the oxygen and nitrogen atoms on the UIO-66@Cr-MIL-101 surface. This type of interaction is particularly important for adsorbing AMX in aqueous solutions. (3) π–π interactions: the benzene ring in amoxicillin can also interact with the aromatic rings of the organic linker molecules in UIO-66@Cr-MIL-101 through π–π interactions. This type of interaction is weaker than electrostatic interactions and hydrogen bonding, but it can still contribute to the overall adsorption capacity of UIO-66@Cr-MIL-101 for AMX.

### Optimization and validation

Following the adjustment of the fitting model, the CCRSM optimization target was used to determine the optimal values of the independent variables, aiming to achieve the desired Re% for AMX adsorption. Based on software predictions for the UIO-66@Cr-MIL-101 nanohybrid, a Re of 99.50% could be attained under the following conditions: pH = 6, contact time of 20 min, 20% loading of MIL-101 NPs on UIO-66 NPs, and an initial concentration of AMX at 80 mg L^−1^. Additionally, the desirability was determined to be 1.0.

## Adsorption isotherm

**Figure 9:**
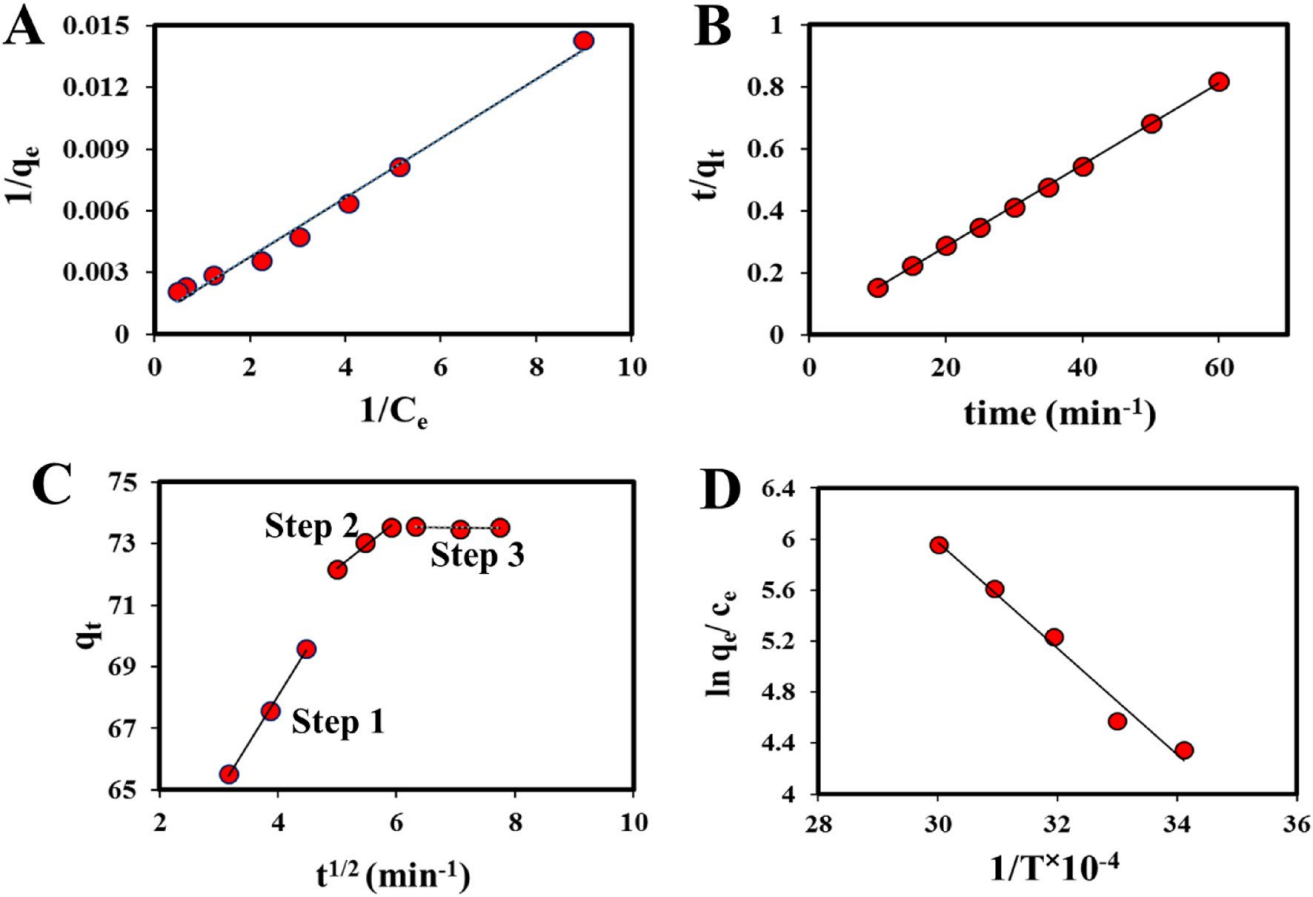
(**A**) The plot of C_e_/q_e_ versus C_e_ of AMX adsorption by UIO-66@Cr-MIL-101 nanohybrid; (**B**) The plot of t/q_t_ versus C_e_ of AMX adsorption by UIO-66@Cr-MIL-101 nanohybrid; (**C**) Three steps of the intraparticle diffusion model for AMX adsorption on UIO-66@Cr-MIL-101 nanohybrid; (**D**) The plot of ln q_e_/c_e_ versus 1/T of AMX adsorption by UIO-66@Cr-MIL-101 nanohybrid.

To analyze the experimental data from AMX removal by UIO-66@Cr-MIL-101 nanohybrid, some famous adsorption isotherms were chosen, as an example, Freundlich^64^, Langmuir^65^ (Fig. 9A), Dubinin Radushkevitch (D-R)^61^, and Temkin^66^.

The Langmuir adsorption isotherm postulates that the adsorption process follows a monolayer mechanism, with adsorption sites assumed to be homogeneous^21^. The linear equation representing this model is provided as follows (Eq. 5).

5 $$\frac{1}{{q}_{e}}=\frac{1}{{q}_{m}}+\frac{1}{{q}_{m}{K}_{l}{c}_{e}}$$

where ${q}_{e}$ and ${q}_{m}$ (mg g^−1^) is the equilibrium adsorption capacity of gas and the maximum theoretical adsorption capacity, respectively. K_L_ (L mg^−1^) represents Langmuir adsorption constant and C_e_ represents equilibrium concentration (mg L^−1^) presented in Table 8. The Langmuir model exhibited a high correlation coefficient (R^2^) of 0.98, indicating a good fit. Furthermore, the maximum adsorption capacity (q_m_) was calculated to be 1111.11 mg g^−1^. These findings confirm that the UIO-66@Cr-MIL-101 nanohybrid is a suitable candidate for AMX removal, as supported by the calculated values.

**Table 8:** Isotherm constant and correlation coefficients calculated for AMX adsorption by UIO-66@Cr-MIL-101 nanohybrid.

Model	Parameter			
Langmuir	q_m_ (mg g^−1^)	K_l_ (L mg^−1^)	R^2^	
	1111.11	0.64	0.98	
Freundlich	n	K_f_ (L mg^−1^)	R^2^	
	1.58	5.9	0.93	
Temkin	B_1_	K_T_ (L mg^−1^)	R^2^	
	259.33	13.01	0.96	
D–R	β(KJ^2^ mmol^−2^)	Q_m_(mg g^−1^)	R^2^	E (KJ mmol^−1^)
	0.063	468.177	0.97	2.806

Freundlich is the other adsorption isotherm model, which is commonly used to describe the adsorption behavior of heterogeneous and non-ideal adsorbents (Eq. 6).

6 $${{\text{Logq}}}_{{\text{e}}}={{\text{LogK}}}_{{\text{f}}}+\frac{1}{{\text{n}}}{{\text{LogC}}}_{{\text{e}}}$$

where K_f_ and n are the Freundlich adsorption constants and the deviation from the ideal homogenous surface, respectively^61^. Based on the calculations, R^2^ was 0.94, and the adsorption process is physical since the n value was 1.58 (n > 1) (Table 8). Moreover, the linear Temkin equation was employed to analyze the experimental adsorption data of AMX onto the UIO-66@Cr-MIL-101 nanohybrid. The corresponding linear equation for the Temkin model is as follows:

7 $${{\text{q}}}_{{\text{e}}}= {{\text{B}}}_{1}{{\text{lnK}}}_{{\text{T}}}+{{\text{B}}}_{1}{{\text{lnC}}}_{{\text{e}}}$$

where ${{\text{K}}}_{{\text{T}}}$ and B_1_ represent the adsorbate’s binding energy and adsorbent and the heat of adsorption, respectively. T(K) is the absolute temperature, and R (8.314 J K^−1^ mol^−1^) is the universal gas constant (Table 8).

The D–R model is employed as the final model for analyzing the adsorption isotherm. Utilizing this model, various parameters, such as the free energy, uptake loading of the adsorbent, and the enhancement of adsorption due to porosity were calculated. Below is the equation that represents the D–R (Dubinin–Radushkevich) model.

8 $${{\text{ln}}({\text{q}}}_{{\text{e}}})={{\text{ln}}({\text{q}}}_{{\text{m}}})-\upbeta {\upvarepsilon }^{2}$$

The calculation of the intercept of the ln q_e_ vs. ε^2^ plot and the slope, provided in Table 8, allows for the determination of the values for β and q_m_ in the equation. To provide an explanation for the average adsorption reaction energy (E) in the D–R model, consider the following^61^:

9 $${\text{E}}= \frac{1}{{(2\upbeta )}^\frac{1}{2}}$$

where E (KJ mmol^−1^) and β (KJ^2^ mmol^−2^) are attributed to the average energy of adsorption and the energy of adsorption, respectively.

Moreover, Polanyi potential is computed by Eq. (10):

10 $$ \upvarepsilon  = {\text{RTln}}\left( {1 + \frac{1}{{{\text{C}}_{{\text{e}}} }}} \right) $$

In this equation R denotes the ideal gas constant, C_e_ shows the equilibrium concentration, and T is the temperature (K).

Based on the analysis of the experimental data provided in Table 8, it is evident that the R^2^ value associated with the D–R isotherm model is relatively lower compared to the other isotherm models. This finding suggests that the D–R model is inadequate for accurately explaining the experimental data in the context of this specific research. Consequently, based on the data presented in Table 8, It can be concluded that the fitting degree to the UIO-66@Cr-MIL-101 nanohybrid exhibits the following order: Langmuir > D–R > Temkin > Freundlich.

## The adsorption kinetic process

In order to gain a comprehensive understanding of the adsorbent's applicability and the underlying adsorption mechanism, a thorough investigation of adsorption kinetics was conducted. Various kinetic models, such as the pseudo-first-order (PFO) (Lagergren and Svenska)^61^, pseudo-second-order (PSO) (Ho and McKay)^67^, and Elovich (Elovich and Larionov)^68^, were employed in this study. These models were utilized to analyze the kinetic data and provide insights into the kinetic mechanisms involved in the adsorption process.

The first-order kinetic model^67^, represented by Eq. (11), is commonly employed to depict liquid/solid adsorption reactions.

11 $${\text{log}}\left({{\text{q}}}_{{\text{e}}}-{{\text{q}}}_{{\text{t}}}\right)={{\text{logq}}}_{{\text{e}}}-\frac{{{\text{K}}}_{1}\cdot {\text{t}}}{2.303}$$

where q_e_ (mg g^−1^) and q_t_ (mg g^−1^) represent the equilibrium and adsorption capacity at time (t), respectively, and k_1_ (min^−1^) denotes the PFO rate constant (Table 9).

**Table 9:** Adsorption kinetic parameters for AMX adsorption onto UIO-66@Cr-MIL-101 nanohybrid.

Model	Parameter		
PFO	R^2^	K_1_ (min^−1^)	Q_e,Calc_ (mg g^−1^)
	0.91	0.02	29.51
PSO	R^2^	K_2_ (min^−1^)	Q_e, Calc_ (mg g^−1^)
	0.99	0.02	52
Elovich	R^2^	α (mg g^−1^ min^−1^)	β(mg g^−1^)
	0.87	1.27E + 08	0.203
Intraparticle	R^2^	K_dif_ (L min^−1^)	C
Step (1)	0.99	3.08	55.71
Step (2)	0.98	1.525	64.58
Step (3)	0.88	− 0.023	73.69
Q_e,Exp_ (mg g^−1^)	73		

The following equation, derived from the PSO model^69^, is utilized to characterize the kinetic of adsorption:

12 $$\frac{{\text{t}}}{{{\text{q}}}_{{\text{t}}}}=\frac{1}{{{\text{K}}}_{2}{{\text{q}}}_{{\text{e}}}^{2}}+\frac{{\text{t}}}{{{\text{q}}}_{{\text{e}}}}$$

The plot $\frac{{\text{t}}}{{{\text{q}}}_{{\text{t}}}}$ versus t computed the amount of ${{\text{K}}}_{2}$, ${{\text{q}}}_{{\text{e}}}$, and R^2^, the results are presented in Table 9 and Fig. 9B. Based on the reported data in Table 9, it can be inferred that the PSO model exhibits an excellent fit for the experimental results. This finding suggests that the sorption of AMX onto the UIO-66@Cr-MIL-101 nanohybrid predominantly follows the PSO kinetic model.

The Elitch kinetic model^70^, as represented by Eq. (13), is employed to elucidate the chemical adsorption behavior between the adsorbent and the adsorbate.

13 $${{\text{q}}}_{{\text{t}}}=\frac{1}{\upbeta }{\text{ln}}({\upalpha \upbeta })+\frac{1}{\upbeta }{\text{ln}}({\text{t}})$$

where $\beta , \alpha ,$ ${\text{and\;q}}_{{\text{t}}}$[mg (g min)^−1^] represent the coverage of the surface and the activation energy range, the initial AMX adsorption rate (g mg^−1^) during any one experiment, and the AMX adsorption capacity of the adsorbent at time t, respectively. Analysis of the parameters presented in Table 9 leads to the conclusion that the Elovich model was inadequate in describing the kinetics of the adsorption process.

The kinetic model employed in this study to examine the kinetics of AMX adsorption by UIO-66@Cr-MIL-101 nanohybrid is the intraparticle diffusion model proposed by Yang et al.^71^. This model elucidates the adsorption mechanism by describing the transfer of the sorbate to the surface and pores of the adsorbent, which is regarded as a critical step in the overall adsorption process (Fig. 9C). Particle diffusion plays a vital role in determining the rate of adsorption during this step, as outlined by Halsey^72^. Equation (14) represents the linear representation of this kinetic model.

14 $${{\text{q}}}_{{\text{t}}}={{\text{K}}}_{{\text{dif}}}{{\text{t}}}^\frac{1}{2}+{\text{C}}$$

In this equation, K_dif_ (mg g^−1^ min^−0.5^), and C are the rate of intraparticle diffusion controlled sorption constant and the intercept, respectively. It should be noted that while the plot of q_t_ versus t^1/2^ passes through the origin, the C parameter is equalized to zero^61^.

The results obtained from this study suggest that the key factor governing the adsorption kinetics is the diffusion model within the particle. The significant deviation observed in the R^2^ values (Table 9) for the adsorption of AMX in UIO-66@Cr-MIL-101 nanohybrid indicates that the chosen model is unsuitable for interpreting the adsorption kinetics. Consequently, the involvement of a speed-restricting step in the uptake process can be rejected.

The AMX adsorption process involves a series of distinct stages, namely: (1) pore diffusion or intraparticle diffusion, (2) film diffusion, (3) mass diffusion, and (4) AMX adsorption occurring on the surface of the adsorbent^73^. Previous literature shows that these graphs are multi-linear^74^, showing the incidence of two or more stages in the process of adsorption.

1. The initial stage of instant adsorption and external surface adsorption.
2. The subsequent phase is characterized by slow adsorption, where rapid diffusion occurs within the particle.
3. The final equilibrium stage is marked by a gradual decrease in diffusion within the particle due to the absence of solute concentration in the mixture.

Based on the kinetic studies carried out on the adsorption of AMX by UIO-66@Cr-MIL-101 nanohybrid, it is proposed that the quasi-second-order model is more suitable for fitting the experimental data. The sequence of R^2^ agreement with UIO-66@Cr-MIL-101 nanohybrid is described: PSO > PFO > Elovich > intraparticle diffusion.

## Adsorption thermodynamics

The following equations were utilized to compute the free energy change of ln K and Gibbs in the adsorption procedure^75^:

15 $${\text{lnK}}^\circ =\frac{qe}{Ce}$$

16 $$\Delta {\text{G}}^\circ =-{\text{RTlnK}}^\circ $$

where K°, R (8.3145 J mol^−1^ K^−1^), and T (K) represent constant balance, the global gas constant, and temperature, respectively. The amount of the $\Delta {\text{G}}^\circ$ is recognized to be negative; thus, this means that the adsorption of AMX by UIO-66@Cr-MIL-101 nanohybrid is automatically procedure in the Van't Hoff equation (Eq. 21). It should be noted that the computation of standard enthalpy changes (∆H°) and entropy changes (∆S°) for absorption involves determining the intercept of lnK° versus 1/T and the slope (Fig. 9D)^66^.

17 $${\text{lnK}}^\circ =\frac{\Delta {\text{S}}^\circ }{{\text{R}}}-\frac{\Delta {\text{H}}^\circ }{{\text{RT}}}$$

Also, it should be noted that the adsorption in this system is exothermic since the amount of the $\Delta {\text{H}}^\circ$ for UIO-66@Cr-MIL-101 nanohybrid is negative (Table 10)^76^.

**Table 10:** Thermodynamic parameters for AMX adsorption onto UIO-66@Cr-MIL-101 nanohybrid.

Adsorbent	Parameter					
UIO-66@Cr-MIL-101		293.15	303.15	313.15	323.15	333.15
	K°	4.347	4.575	5.236	5.612	5.955
	ΔG° (kJ mol^−1^)	− 1.596	− 1.153	− 1.363	− 1.508	− 1.649
	ΔH° (kJ mol^−1^)	− 0.526				
	ΔS° (kJ mol^−1^)	0.216				

Furthermore, as the temperature increases, there is a noticeable reduction in the magnitude of ΔG°, indicating a declining trend in the AMX uptake on the UIO-66@Cr-MIL-101 nanohybrid at elevated temperatures. Additionally, the positive value of ΔS° leads to an increase in the degrees of freedom for adsorbed molecules on the surface, resulting in an enhancement of the interconnection between the solution and solid during the adsorption process by UIO-66@Cr-MIL-101 nanohybrid.

## Comparison of the q_m_ of UIO-66@Cr-MIL-101 nanohybrid of this work with other adsorbents

In this study, a very inexpensive adsorbent with easy and rapid synthesis was used to remove AMX. The results showed a significant amount of q_m_ for the removal of AMX by UIO-66@Cr-MIL-101 nanohybrid compared to other adsorbents in the literature (Table 11).

**Table 11:** Comparison of the q_m_ of UIO-66@Cr-MIL-101 nanohybrid found in the literature for AMX removal.

Sorbent	Kinetic	Isotherm	q_m_ (mg g^−1^)	Refs.
UIO-66@Cr-MIL-101	PSO	Langmuir	1111.11	This work
UIO-66	PSO	Langmuir	723	^77^
MOF-5	PSO	Freundlich	662	^78^
MIL-53(Al)	PSO	Langmuir	467	^79^
PCN-124-stu(CU)	Redlich-P	Langmuir	198	^80^
ZIF-8 derived NPC	PSO	Freundlich	416	^81^
[Zn_6_(IDC)_4_(OH)_2_(Hprz)_2_]_n_ (IDC ¼ planar imidazole-4,5-dicarboxylate; prz ^¼^ piperazine)	PFO	Langmuir	185	^82^

## Study of regeneration

**Figure 10:**
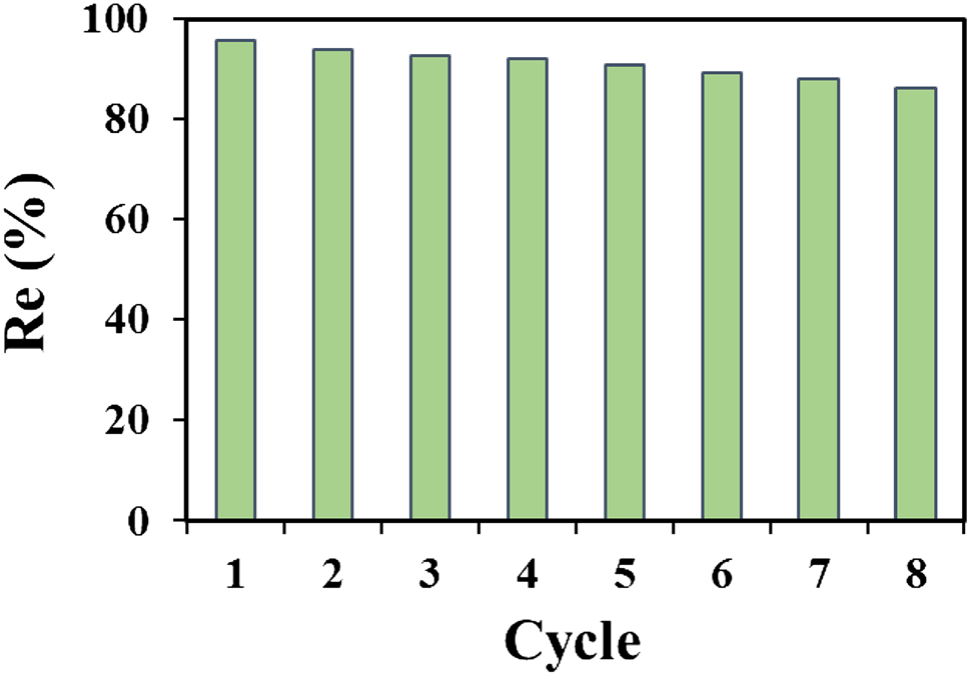
Reusability of UIO-66@Cr-MIL-101 nanohybrid by adsorption/desorption process for eight consecutive cycles.

The stability and reusability of sorbents are important factors for the widespread application of them^83^. Thus, in order to analyze the durability of the UIO-66@Cr-MIL-101 nanohybrid, the adsorption–desorption recycling test was employed (Fig. 10). After each cycle of adsorbent application, AMX desorption from UIO-66@Cr-MIL-101 nanohybrid was performed by being washed with ethanol and utilized for the following cycle. As can be seen, there is negligible loss of adsorption sites even after undergoing eight cycles. This remarkable level of stability and durability exhibited by the UIO-66@Cr-MIL-101 nanohybrid highlights its potential for various applications in adsorption processes, all while maintaining environmental integrity. The exceptional repeatability demonstrated by the nanohybrid highlights its potential for diverse applications in adsorption processes. Its environmentally friendly nature further contributes to minimizing pollution, making it an attractive solution for sustainable and eco-friendly practices.

## Conclusion

In this study, the removal of AMX antibiotic from wastewater was investigated by UIO-66@Cr-MIL-101 nanohybrid (through adsorption process), and it was observed that this nanoadsorbent has an excellent adsorption capacity to remove this AMX (1111.11 mg g^−1^). CCRSM was used to optimize four parameters: loading percentage Cr-MIL-101 NPs, initial concentration of AMX, contact time, and pH parameters. The results of the study showed that it follows the quadratic model. The Re% of AMX is equal to 99.50% and was obtained under the following conditions: the loading value of 20% Wt%, the initial concentration of AMX 80 mg L^−1^, contact time 20 min, and pH = 6. Also, the study of adsorption isotherm and kinetics shows that the Langmuir isotherm model and PSO kinetics successfully describe the equilibrium adsorption data. Investigation of thermodynamics of adsorption showed that the adsorption of AMX by UIO-66@Cr-MIL-101 nanohybrid is spontaneous and exothermic under optimal experimental conditions. Therefore, this research shows that the synthesized nanohybrid can be considered a new and excellent adsorbent due to its high adsorption capacity and short equilibrium time.

## Data Availability

All data generated or analyzed during this study are included in this published article.
